# Exploring the association between dietary Inflammatory Index and chronic pain in US adults using NHANES 1999–2004

**DOI:** 10.1038/s41598-024-58030-w

**Published:** 2024-04-16

**Authors:** Lunxue Qing, Yingying Zhu, Changhe Yu, Yang Zhang, Jinxia Ni

**Affiliations:** 1 The First Clinical Medical College, Beijing University of Chinese Medicine, Beijing, China; 2 Dongzhimen Hospital, Beijing University of Chinese Medicine, 5 Haiyuncang, Dongcheng District, Beijing, 100700 China

**Keywords:** Persistent pain, Dietary inflammation potential, Dose–effect relationship study, Prevalence study, National health and nutrition examination survey, Neuropathic pain, Epidemiology, Risk factors

## Abstract

Chronic pain, a substantial public health issue, may be influenced by dietary patterns through systemic inflammation. This cross-sectional study explored the association between Dietary Inflammatory Index (DII) and chronic pain among 2581 American adults from NHANES data. The DII, ranging from − 4.98 to 4.69, reflects the inflammatory potential of the diet, with higher scores indicating greater pro-inflammatory capacity. Our findings showed no significant association between the continuous DII score and chronic pain prevalence. However, a nonlinear relationship emerged. When the DII was categorized, a significant association between higher DII scores (DII ≥ 2.5) and chronic pain prevalence was observed. The analysis uncovered a U-shaped pattern, with an inflection point at a DII score of − 0.9, indicating an association between both low and high levels of dietary inflammation are associated with higher pain prevalence. This nuanced interaction between dietary inflammation and chronic pain indicates the possibility of incorporating dietary modification into pain management strategies and underscores the need for further research into the long-term effects of diet on chronic pain.

## Introduction

Chronic pain, as defined by the International Association for the Study of Pain (IASP), is pain that persists or recurs for more than three months and poses a significant healthcare challenge^1^. Historically, research into chronic pain has transitioned from viewing it solely as a symptom of other conditions to recognizing it as a standalone health issue, especially in the case of chronic primary pain. This shift is evident in the International Classification of Diseases, which categorizes chronic pain into primary and secondary syndromes^1–3^. Primary chronic pain, unlike secondary chronic pain, is not a symptom of an underlying disease but a separate condition often lacking identifiable causes. This understanding marked a pivotal change in how pain is perceived and managed in clinical settings. This issue is estimated to affect approximately 20% of the global population, according to recent studies^4,5^, and imposes a significant burden on healthcare systems, leading to an increase in medical consultations and associated costs^6,7^. Chronic pain arises from a complex interplay of physical and psychological factors, potentially leading to irreversible changes in the nervous system over time^8^. The evolution of chronic pain research reflects a growing recognition of its complexity and the need for multidisciplinary approaches to its management. Early detection and timely intervention have become crucial approaches in managing chronic pain.

Recent research highlights the pivotal role of the immune system in chronic pain development, indicating that dietary factors may influence this process through immune system modulation^9–12^. Dietary components can regulate the relationship between pain and inflammation, offering new avenues for pain relief^13^. An individual's daily dietary habits are indicative of their risk for inflammation and oxidative stress^14^. Systemic inflammation, influenced by the central nervous system, alters pain perception. Simultaneously, inflammation outside the central nervous system can increase pain sensitivity^15^. The intake of essential minerals, such as magnesium and zinc, has been associated with a reduction in inflammation-related pain^16–18^. Conversely, a review indicates that obesity-related dietary patterns, such as a high-fat diet, primarily enhance nociception by increasing inflammation and recruiting M1 macrophages that trigger immune responses^19^. Another study has observed excessive calorie consumption and diets high in sugar, fat, sodium, and caffeine among chronic pain patients undergoing long-term opioid therapy^20^.

The Dietary inflammatory index (DII) measures an individual's potential for dietary inflammation. Evidence suggests that a high DII is associated with an increased risk of mortality from all causes, cancer, and cardiovascular disease, and it is also associated with an elevated risk of experiencing musculoskeletal and neurological conditions, such as joint pain, headaches, or migraines^21^. DII is designed to reflect all evidence from diverse populations using different research designs and dietary assessment methods^22^. Despite having a lower weighting, the DII also incorporates evidence from experimental animals and cell cultures^23^. Studies have shown that a high DII score is significantly related to BMI, hypertension and diabetes^24,25^. Another study shows that DII is positively related to the inflammatory level of Japanese men, but this correlation is limited in women^26^. Its widespread use in studies shows its effectiveness in examining the link between diet-related inflammation and diseases like heart conditions and certain cancers. The DII focuses on the nutritional value of food rather than just categorizing food types^23,27,28^.

While some evidence suggests a connection between diet-induced inflammation and chronic pain^29–31^, conclusive evidence is not yet established. Many studies have addressed the role of dietary factors in body muscle pain; however, the specific link between dietary inflammatory potential and chronic pain remains under-explored. Elucidating the association between the DII assessment of dietary inflammation and chronic pain is imperative. This cross-sectional study aims to examine the association between the DII and the prevalence of chronic pain among American adults. The findings may contribute to a greater understanding of how diet-related inflammation correlates with chronic pain, potentially guiding future longitudinal research that could inform dietary recommendations for chronic pain management.

## Methods

### Study design

This study assessed the association between the dietary inflammatory index and chronic pain in individuals aged 20 and above, utilizing data from three NHANES cycles (1999–2004). NHANES, a program of studies designed to evaluate the health and nutritional status of adults and children in the United States, employs a rigorous multi-stage probabilistic sampling methodology to ensure national representativeness. The study protocols adhere to the Declaration of Helsinki and have been ethically approved by the NCHS Research Ethics Review Board, with all adult participants providing written informed consent. Our secondary analysis follows the STROBE guidelines for cross-sectional studies^32^, and did not necessitate additional institutional review board approval^33^. Comprehensive details on NHANES' methodology and ethics can be found on the CDC and NCHS website (https://www.cdc.gov/nchs/nhanes/index.htm).

In this analysis, we initially reviewed data from 31,126 participants collected during the NHANES cycles from 1999 to 2004. We narrowed our focus to adults aged 20 years and above, which included 15,332 individuals. From this subset, we excluded 11,754 subjects due to missing chronic pain data, 351 subjects for incomplete dietary records, 556 subjects lacking crucial data related to our study variables, and 90 pregnant women. After applying these exclusion criteria, our final analytic sample consisted of 2,581 adults. This sample was composed of 1,540 individuals reporting chronic pain and 1,041 individuals without chronic pain.

### Definition of chronic pain

Chronic pain is pain lasting or recurring for over three months. We identified chronic pain sufferers using the Miscellaneous Pain Questionnaire (MPQ110), which asked about pain duration (How long have you experienced this pain?). Specifically, those indicating pain for "at least three months but less than one year" or "greater than one year" were classified as having chronic pain. Responses of "less than a month" or "At least one month but less than three months" meant no chronic pain. Non-responders or "I don't know" answers were treated as missing values^34–36^.

### Calculation of DII

The DII was derived using a method established by Shivappa et al.^23^. It encompasses 27 dietary components from NHANES, including carbohydrates, protein, total fat, alcohol, fiber, cholesterol, saturated fat, monounsaturated fatty acids (MUFAs), polyunsaturated fatty acids (PUFAs), niacin, folic acid, β-carotene, caffeine, energy, n-3 fatty acids, n-6 fatty acids, micronutrients such as vitamins (A, B1, B2, B6, B12, C, E) and minerals (Fe, Mg, Zinc, Selenium). A negative DII score indicates an anti-inflammatory diet, while a positive score suggests a pro-inflammatory diet. According to the methods of Shivappa et al.^23^, the DII calculation is based on a global database, which provides a 'global daily mean intake’ and ‘standard deviation’ for each food parameter. The database was built upon the analysis of relationships between food components and inflammation, as found in 1943 published articles. The 'global daily mean intake’ and ‘standard deviation’ are used to calculate a Z-score for each individual's intake. This Z-score is obtained by subtracting the 'standard mean' from the reported intake amount and dividing by its standard deviation^23^. To adjust for potential right skewing in the data, the resulting value is then transformed into a percentile score. For a symmetric distribution centered around 0 (indicating no inflammation) and bounded between − 1 (maximally anti-inflammatory) and + 1 (maximally pro-inflammatory), each percentile score is doubled and then reduced by 1. This centered percentile value for each dietary parameter is then multiplied by its respective 'overall food parameter-specific inflammatory effect score' to derive the 'food parameter-specific DII score.' Finally, all of these 'food parameter-specific DII scores' are aggregated to determine the 'overall DII score' for each individual. For this calculation, 27 specific dietary components were utilized. Notably, this did not include data on spices and flavonoids, as these components were not captured in the 24-h dietary recall data of NHANES 1999–2004. According to prior studies, DII scores computed with 25–30 dietary components generally range between − 5.5 and + 5.5^22^. It has been successfully applied in numerous studies and can be regarded as a reliable tool for assessing diet-related inflammation^37–40^. This comprehensive approach ensures an accurate and representative assessment of the inflammatory potential of an individual's diet.

### Covariates

The analysis considered several covariates encompassing socio-demographic factors (age, gender, race/nationality, marital status, education level, family income), lifestyle factors (physical activity, smoking status), and concurrent diseases (coronary heart disease, stroke, hypertension, diabetes). Additionally, we examined dietary supplement use and measured C-reactive protein (CRP) levels. Race/ethnicity was classified into non-Hispanic whites, non-Hispanic blacks, Mexican Americans, or other racial backgrounds. Marital status was divided into married, cohabiting with partners, or living alone. Education level was stratified into three categories: less than nine years, 9 to 12 years, and more than 12 years. Household income, based on the poverty-income ratio (PIR), was categorized into three groups: low (PIR ≤ 1.3), medium (PIR > 1.3 and PIR ≤ 3.5), and high (PIR > 3.5). Physical activity was classified as sedentary, moderate (entailing exercise for at least 10 min in the past 30 days, resulting in mild sweating or breathing), and intense (entailing exercise for at least 10 min in the past 30 days, resulting in significant sweating or an increased heart rate). Smoking status included never smokers (having smoked fewer than 100 cigarettes), former smokers (having quit after smoking more than 100 cigarettes), and current smokers. Body mass index (BMI) was computed based on weight and height. Conditions such as coronary heart disease, stroke, hypertension, and diabetes were identified based on participants' self-reporting of a doctor's diagnosis in the questionnaire. CRP levels were quantified using high-sensitivity latex-enhanced nephelometry on a BNII nephelometer (Dade Behring Diagnostics, Inc., Newark, DE, USA)^41^.

### Statistical analysis

Categorical variables were presented as percentages. Continuous variables were presented as means (standard deviation, SD) for normally distributed data and medians (interquartile range, IQR) for non-normally distributed data. Group comparisons involved one-way ANOVA for normally distributed continuous variables, the Kruskal–Wallis test for skewed continuous variables, and the chi-square test for categorical variables. Logistic regression models were employed to investigate the relationship between DII and chronic pain. Model 1 involved adjustments for age and gender. Model 2 extended these adjustments to include socio-demographic characteristics, such as race and marital status. Model 3 further adjusted for lifestyle factors like smoking and physical activity, and concurrent diseases such as hypertension and diabetes. We employed Restricted Cubic Spline (RCS) regression to model the dose–response relationship between the dietary inflammatory index and chronic pain, utilizing specific percentiles (5th, 35th, 65th, and 95th) for its flexibility in capturing nonlinear associations after adjusting for covariates in Model 3.

We conducted stratified analyses to examine the interaction between the dietary inflammatory index and the prevalence of chronic pain across various subgroups. These analyses included stratification by sex, body mass index (BMI < 25 vs. ≥ 25 kg/m^2^), hypertension status, and diabetes. Within each subgroup, we adjusted for all other covariates to isolate the specific effect of each stratification variable on the relationship between dietary inflammatory index and chronic pain. Multivariate logistic regression was used to assess subgroup heterogeneity, and likelihood ratio tests were conducted to examine interactions between subgroups and the dietary inflammatory index. In a sensitivity analysis to ensure the robustness of our findings, we excluded participants with an unusual energy intake, specifically those consuming less than 500 kcal/day and more than 5000 kcal/day (n = 81). This exclusion was performed to minimize the potential influence of outliers on the results. We didn't estimate a priori statistical power because the data provided only determined the sample size. All analyses were conducted using the stratification and weighting scheme recommended by the National Center for Health Statistics for NHANES^42^. The analyses were performed using R statistical software version 4.1.2 (https://www.r-project.org, accessed September 16th, 2023) and Free Statistics Software Version 1.9^43^, with significance set at *p* < 0.05.

## Results

### Study population

**Figure 1:**
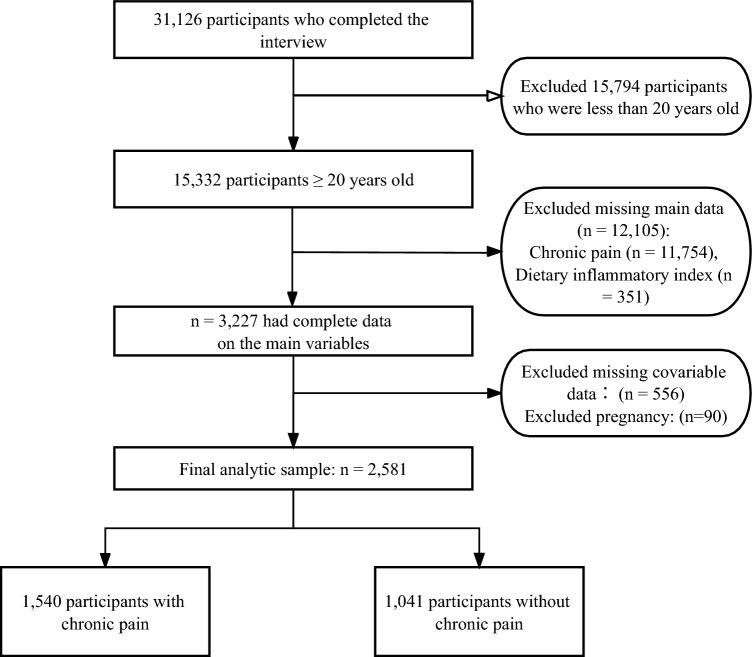
Flowchart of study selection.

Out of 31,126 participants interviewed, 15,794 were under 20 years old. Exclusions included individuals with missing chronic pain data (n = 11,754), incomplete diet records (n = 351), absent covariate data (n = 556), and pregnancy (n = 90). Consequently, this cross-sectional study included 2581 participants from NHANES (1999–2004). Figure 1 provides a comprehensive visualization of the inclusion and exclusion processes.

### Baseline characteristics

Baseline characteristics are illustrated in Table 1, categorized by dietary inflammatory index quartiles. Of the subjects, 58.29% reported chronic pain. On average, participants were 46.03 years old (SD = 15.09), with 54.47% being women. Those with higher dietary inflammatory indices tended to be older, female, living alone, current smokers, lower education, lower income, less physical activity, and a history of hypertension, diabetes, and stroke. They were also less likely to use dietary supplements and had higher C-reactive protein levels. Table S1 presents a comparison of baseline characteristics between subjects (≥ 20 y) included in the study and those excluded. Table S2 displays the distribution of intake for the 27 food components used to calculate the DII, along with the corresponding intake distribution of pro-inflammatory and anti-inflammatory food items. This supplementary table elucidates any potential differences in demographic and health-related variables, ensuring transparency regarding the representativeness of the study sample.

**Table 1:** Population characteristics by categories of dietary inflammatory index.

Variables	Total	Dietary inflammatory index				*p*
		Q1 (− 4.98 to − 0.2)	Q2 (− 0.21 to 1.3)	Q3 (1.31–2.49)	Q4 (2.5–4.69)	
NO	2671	667	667	665	672	
Age(year), Mean (SD)	46.03 (15.09)	46.42 (14.90)	46.46 (14.82)	45.98 (15.04)	45.27 (15.57)	0.56
Sex, n (%)						
Male	45.53	64.2	50.31	39.51	27.33	< 0.01
Female	54.47	35.8	49.69	60.49	72.67	
Race/ethnicity, n (%)						
Non-Hispanic white	78.04	81.15	80.42	76.8	73.67	0.04
Non-Hispanic black	8.55	6.37	6.87	9.92	11.13	
Mexican American	4.34	5.23	4.61	3.43	4.03	
Others	9.07	7.25	8.09	9.86	11.17	
Marital status, n (%)						
Married or living with a partner	65.24	67.33	70.89	61.41	61.24	0.05
Living alone	34.76	32.67	29.11	38.59	38.76	
Education level, n (%)						
< 9	5.1	3.85	3.93	5.14	7.51	< 0.01
9–12	41.76	35.1	41.08	44.29	46.9	
> 12	53.14	61.05	54.99	50.57	45.59	
Poverty income ratio, Mean (SD)	2.87 (1.63)	3.26 (1.55)	3.13 (1.60)	2.65 (1.57)	2.44 (1.64)	< 0.01
BMI (kg/m2), Mean (SD)	29.08 (6.97)	28.62 (6.9)	29.16 (6.64)	29.63 (7.16)	28.95 (7.13)	0.28
Physical activity, n (%)						
Sedentary	37.82	29.67	37.36	38.36	46.18	< 0.01
Moderate	30.07	32.42	30.29	28.72	28.71	
Vigorous	32.12	37.91	32.35	32.93	25.11	
Smoking status, n (%)						
Never	43.13	45.44	44.85	40.4	41.69	< 0.01
Former	26.73	35.05	25.28	24.99	21.21	
Current	30.14	19.51	29.86	34.61	37.1	
Hypertension, n (%)	27.67	28.11	26.06	27.8	28.64	0.85
Diabetes, n (%)	8.49	7.92	7.39	8.78	9.86	0.55
Coronary heart disease, n (%)	5.2	4.88	5.94	4.26	5.72	0.61
Stroke, n (%)	3.36	3.89	2.72	2	4.75	0.06
Dietary supplements taken, n (%)	55.05	63.72	53.9	54.64	47.59	< 0.01
CRP (mg/dl), Median (IQR)	0.23 (0.09, 0.55)	0.16 (0.08, 0.32)	0.24 (0.12, 0.58)	0.24 (0.1, 0.57)	0.29 (0.11, 0.66)	< 0.01
Chronic pain, n (%)	58.29	52.61	53.93	61.8	65.03	< 0.01

Abbreviations: BMI: body mass index; CRP: C-reactive protein; IQR: interquartile range; SD: standard deviation.

*Note*: The sample sizes presented in Table 1 are unweighted counts reflecting the actual number of observations. All other results reported in this table are based on weighted data to account for the complex survey design and to ensure representativeness of the study population. The weighting procedure adjusts for potential sampling biases and non-response, allowing for more accurate estimates that are generalizable to the target population.

### Relationship between dietary inflammatory index and chronic pain

**Figure 2:**
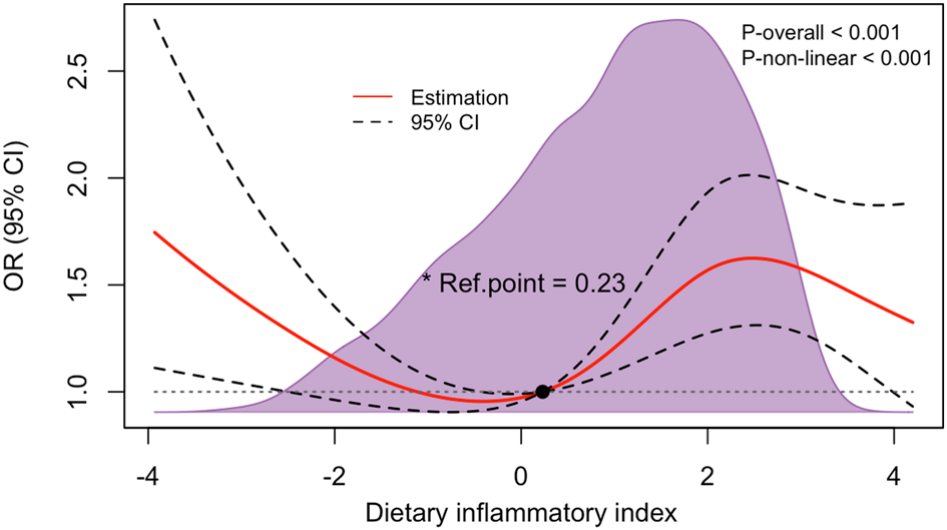
Weighted dose–response association of dietary inflammatory index and chronic pain. *Note*: Solid and dashed lines represent the predicted value and 95% confidence intervals. They were adjusted for age, sex, race, marital status, poverty income ratio, education level, body mass index, physical activity, smoking status, coronary heart disease, stroke, hypertension, diabetes mellitus, dietary supplements taken, and C-reactive protein. *The reference point is established based on the overall median derived from the simulated world dietary inflammatory index.

Table 2 presents the results of univariate logistic regression analysis, which indicates associations between age, race, body mass index, education level, family income, physical activity, smoking, hypertension, diabetes, coronary heart disease, and chronic pain. After adjusting for all confounders, the multivariate logistic regression analysis revealed no significant association between the continuous variable DII and the prevalence of chronic pain (OR 1.05, 95% CI 0.99–1.11, *p* = 0.081). Nevertheless, when categorizing DII, there was a significant association observed in the highest quartile Q4 (≥ 2.5) (OR 1.43, 95% CI 1.08–1.89, *p* = 0.015), indicating a potential nonlinear relationship between DII and chronic pain (Table 3). The RCS curve fitting confirmed this nonlinear relationship (p-nonlinear < 0.001) (Fig. 2). Threshold effect analysis using the likelihood ratio test identified the inflection points in this relationship. Threshold analysis revealed that when the DII ≥ − 0.9, DII positively correlated with chronic pain (OR 1.094, 95% CI 1.018–1.176, *p* = 0.014), indicating a 9.4% increased risk per unit increase. Conversely, when DII < − 0.9, it negatively correlated with chronic pain (OR = 0.767, 95% CI: 0.591–0.994, p = 0.045), implying a 23.3% risk reduction per unit decrease (Table 4).

**Table 2:** Bivariate associations between variables and chronic pain.

Variable	OR (95% CI)	*p* Value	Variable	OR (95% CI)	*p* Value
Age, y	1.03 (1.02–1.04)	< 0.001	Poverty income ratio	0.89 (0.85–0.94)	< 0.001
Sex, n (%)			Body mass index, kg/m^2^	1 (0.99–1.02)	0.674
Male	1 (Ref)		Smoking status, n (%)		
Female	1.12 (0.85–1.47)	0.417	Never	1 (Ref)	
Race/ethnicity, n (%)			Former	1.37 (1.1–1.7)	0.006
Non-Hispanic white	1 (Ref)		Current	1.68 (1.27–2.22)	< 0.001
Non-Hispanic black	1 (0.78–1.28)	0.986	Physical activity, n (%)		
Mexican American	0.69 (0.47–1.01)	0.058	Sedentary	1 (Ref)	
Others	0.82 (0.61–1.11)	0.197	Moderate	0.67 (0.51–0.87)	0.003
Education level (years), n (%)			Vigorous	0.39 (0.3–0.5)	< 0.001
< 9	1 (Ref)		Coronary heart disease, n (%)	1.85 (1.12–3.06)	< 0.001
9–12	0.89 (0.6–1.32)	0.55	Stroke, n (%)	1.37 (0.74–2.51)	0.307
> 12	0.66 (0.42–1.03)	0.068	Diabetes, n (%)	1.98 (1.40–2.81)	< 0.001
Marital status, n (%)			Hypertension, n (%)	1.7 (1.34–2.14)	< 0.001
Married or living with a partner	1 (Ref)		Dietary supplements taken, n (%)	1.09 (0.92–1.28)	0.308
Living alone	0.77 (0.61–0.97)	0.026	C-reactive protein, mg/dl	1.09 (0.94–1.26)	0.261
Dietary inflammation index	1.09 (1.03–1.16)	0.003			

Abbreviations: OR: odds ratio; CI: confidence interval; Ref: reference.

*Note*: Results are based on weighted data.

**Table 3:** Association between dietary inflammatory index and chronic pain in multiple regression model.

Outcome	Total	Chronic pain, %	Crude model		Model 1		Model 2		Model 3	
			OR (95% CI)	*p* Value	OR (95% CI)	*p* Value	OR (95% CI)	*p* Value	OR (95% CI)	*p* Value
DII	2671	58.29	1.09 (1.03–1.16)	0.003	1.1 (1.04–1.16)	< 0.001	1.08 (1.02–1.14)	0.009	1.05 (0.99–1.11)	0.081
DII (quartile)										
Q1	667	52.61	1(Ref)		1(Ref)		1(Ref)		1(Ref)	
Q2	667	53.93	1.05 (0.74–1.5)	0.762	1.06 (0.78–1.45)	0.707	1.02 (0.75–1.4)	0.887	0.95 (0.7–1.29)	0.719
Q3	665	61.8	1.46 (1.05–2.02)	0.024	1.51 (1.1–2.09)	0.013	1.41 (1.02–1.96)	0.041	1.32 (0.94–1.86)	0.101
Q4	672	65.03	1.67 (1.23–2.28)	0.002	1.8 (1.34–2.41)	< 0.001	1.63 (1.23–2.17)	0.001	1.43 (1.08–1.89)	0.015
Trend test				< 0.001		< 0.001		< 0.001		0.004

Abbreviations: CI: confidence interval; DII: dietary inflammatory index; OR: odds ratio; Ref: reference.

*Note*: This table presents the results from a multivariable regression analysis, adjusted for potential confounders. All analyses have been weighted to account for the survey's complex sampling design, ensuring that the findings are representative of the population studied.

Q1 (≤ − 0.2), Q2 (− 0.21–1.30), Q3 (1.31–2.49), Q4 (≥ 2.5).

Model I: Adjusted for age and sex.

Model II: Adjusted for age, sex, race, marital status, poverty income ratio, and education level.

Model III: Adjusted for all these variables, including age, sex race, marital status, poverty income ratio, education level, body mass index, physical activity, smoking status, coronary heart disease, stroke, dietary supplements taken, hypertension, diabetes mellitus and C-reactive protein.

**Table 4:** Threshold effect analysis of the relationship of DII with chronic pain.

DII	Adjusted model	
	OR (95% CI)	*p* value
< − 0.9	0.767 (0.591–0.994)	0.045
≥ − 0.9	1.094 (1.018–1.176)	0.014
Likelihood ratio test		0.018

Abbreviations: CI, confidence interval; DII, dietary inflammatory index, OR, odds ratio.

Note: Adjusted for age, sex, race, marital status, poverty income ratio, education level, body mass index, physical activity, smoking status, coronary heart disease, stroke, hypertension, diabetes mellitus, dietary supplements taken, and C-reactive protein.

Only 99.9% of the data is shown.

### Stratified analysis

Stratified analyses were conducted to assess the influence of various factors on the association between DII and chronic pain, considering sex, BMI, hypertension, and diabetes. The results for these groups revealed no significant interactions (Figure S1).

### Sensitivity analysis

In sensitivity analysis, we excluded individuals with extreme energy intake, resulting in 2,500 individuals. The relationship between DII and chronic pain remained stable. Compared to Q1 (≤ − 0.14), adjusted ORs for DII and chronic pain in Q2 (− 0.15–1.31), Q3 (1.32–2.48), and Q4 (≥ 2.49) were 0.93 (95% CI 0.67–1.28, *p* = 0.636), 1.3 (95% CI 0.92–1.82, *p* = 0.131), and 1.41 (95% CI 1.05–1.9, *p* = 0.023), respectively (Table S3). The nonlinear relationship was found in RCS regression analysis (nonlinear, *p* < 0.001) (Figure S2).

## Discussion

In this comprehensive cross-sectional analysis within a diverse American adult population, our investigation unveiled a complex, U-shaped dynamic between dietary inflammation—as quantified by the DII—and the experience of chronic pain. This relationship was punctuated by a distinctive turning point, underscoring the nuanced interplay between DII and chronic pain. Our results illuminate intriguing patterns that imply dietary components could exert differential influences on inflammation and its symptomatic manifestations. Such insights lay a foundation for prospective longitudinal research, paving the way for dietary modifications as potential therapeutic strategies in the context of chronic pain management.

Prior studies have linked poor dietary patterns to various forms of chronic pain, considering them predictive, persistent, or potential contributors to chronic musculoskeletal pain^44,45^. Emerging evidence underscores the pivotal role of diet in chronic inflammation regulation^46^. individuals suffering from chronic pain frequently demonstrate increased calorie intake and maintain dietary patterns marked by elevated levels of sugar, fat, sodium, and caffeine. It's worth mentioning that some studies have associated a high dietary inflammatory index with BMI^47^. However, our stratified analysis revealed no significant interaction between BMI and DII. Our results align with previous studies suggesting a link between dietary patterns and pain sensitivity^31^. For instance, rheumatoid arthritis patients have reported pain relief through dietary measures like fasting, plant-based, Mediterranean, and elimination diets^48^.

Moreover, the findings from a comprehensive systematic review are noteworthy, revealing an inverse relationship between adherence to healthy dietary patterns, particularly the Mediterranean diet, and noncancer pain prevalence^49^. Complementary evidence from another study further supports this association, demonstrating that elderly individuals who follow the Mediterranean diet may experience enhanced pain relief^50^. However, it's crucial to note that not all studies support these findings. A cross-sectional study among elderly Iranians found no significant correlation between the dietary inflammatory index and musculoskeletal pain^51^. In contrast, a more extensive cohort study in Spain, with a substantial sample size and an average follow-up period of 3.2 years, reported a significant association between an increase in the DII and pain events. This correlation was particularly pronounced in subjects with lower physical activity levels^52^. These divergent findings illustrate the intricate and multifaceted nature of the relationship between diet and pain, reflecting the diversity of study designs, populations, and methodologies in the existing literature. In analyzing the association between the DII and chronic pain, our findings present a U-shaped relationship with an inflection at -0.9. Notably, a lower DII, which suggests an anti-inflammatory dietary pattern, was associated with an increased likelihood of chronic pain. This association, while initially seeming paradoxical, may be explicable through several hypotheses which warrant further investigation.

One plausible explanation could be the modification of dietary habits following the onset of chronic pain, where individuals may adopt anti-inflammatory dietary patterns in response to their condition. However, these dietary changes might not fully capture the complexity of pain etiology or address individual nutrient deficiencies that could exacerbate pain symptoms. Additionally, the DII does not account for all bioactive food compounds and their potential to influence pain perception through mechanisms not solely related to systemic inflammation. Moreover, there may be unmeasured confounding variables, such as psychosocial stressors or genetic predispositions, which are not accounted for by the DII but could significantly impact the experience of pain. These factors may introduce bias in the interpretation of the relationship between diet and pain.

It is also critical to recognize that the DII is an aggregate measure, and individual dietary components within an anti-inflammatory pattern may have diverse effects on pain perception. Certain components, despite their anti-inflammatory properties, might still be associated with an increased pain experience in susceptible individuals.

While the biological mechanisms underpinning the relationship between dietary inflammation and chronic pain require further exploration, existing evidence supports its plausibility. For instance, studies suggest that adopting a low-sugar diet may mitigate migraines by reducing inflammation^29^. Additionally, ample evidence indicates that anti-inflammatory diets are associated with pain relief^49,50,52–56^. Reducing the intake of saturated fats and added sugars may inhibit afferent vagal nerve fibers from detecting pro-inflammatory cytokines associated with Western diets and prevent the transmission of peripheral inflammation signals to the brain^57^. Positive findings from preclinical studies suggest that nutritional interventions could serve as potential inhibitors of neuro-inflammation by potentially reducing the activation of glial cells and subsequent central nervous system sensitization^57^.

Additionally, there is a correlation between diets rich in fat and elevated levels of plasma aminotransferases and pro-inflammatory molecules. These dietary patterns have the potential to hinder the ability of antioxidants while promoting lipid peroxidation, protein oxidation, DNA damage, and the activation of genes or proteins related to the JNK pathway, which has been linked to the occurrence of neuropathic pain^58–60^. Additionally, pro-inflammatory diets may activate TLR 4 signal transduction, a key regulator of pro-inflammatory cytokines, ultimately contributing to neuropathic pain^61–64^. Scientific investigations have established that limonene, an organic substance present in citrus fruits, possesses the ability to mitigate mechanical sensitivity by suppressing pro-inflammatory mediators such as interleukin-1β and tumor necrosis factor^65^. Contrarily, excessive consumption of fat has been associated with heightened nociceptive responsiveness in mice afflicted with knee arthritis^66^.

Our results indicate that either a lower or higher DII may suggest an increased risk of chronic pain. A U-shaped association could be interpreted to suggest an optimal level of DII intake that balances the lowest risk of chronic pain. A cross-sectional study from Iran, involving 125 women aged 20–46, demonstrated that a diet high in salt and sugar, along with Western dietary patterns was positively correlated with the DII. Conversely, a healthy dietary pattern marked by high consumption of eggs, poultry, chicken, legumes, and refined grains was associated with lower levels of the inflammation index^67^. Previous study has shown that participants with higher DII scores consume lower amounts of polyunsaturated fats, monounsaturated fats, and fiber, and higher amounts of saturated fats^68^. A lower DII score is negatively correlated with the consumption of healthy foods and nutrients. Diets rich in pro-inflammatory food parameters, such as saturated fatty acids, and moderately deficient in anti-inflammatory food components, such as fruits and vegetables, can lead to an increase in inflammatory biomarkers, such as IL-6 and homocysteine levels^69^. Considering that muscle strength and quality are crucial protective factors for musculoskeletal chronic pain, adequate intake of proteins, meats, and fats is essential for maintaining muscle mass. Overemphasizing an anti-inflammatory diet could potentially reduce dietary diversity, which is not desirable. Different dietary types offer the possibility of initiating various stimulus patterns at different levels (neurocognitive, emotional, digestive), underscoring the importance of dietary diversity in regulating the emotional and cognitive aspects of chronic pain^70^. Excessively focusing on anti-inflammatory diets may not necessarily reduce chronic pain; it might even increase it. For those with chronic pain, it's essential to maintain a balanced approach to anti-inflammatory diets. The optimal diet should strike a harmonious balance. In seeking a 'harmonious balance,' we propose a dietary approach that emphasizes variety and nutrient adequacy. This approach aligns with the principles of the Mediterranean diet, which has been associated with beneficial health outcomes beyond its anti-inflammatory effects. The integration of a wide range of foods ensures that the diet provides all necessary macro and micronutrients, supporting overall health and potentially reducing the risk of chronic pain^71,72^. It's important to consider that diet is one component of a holistic health strategy, and its optimal composition may vary among individuals. Thus, future research should investigate these relationships longitudinally and in diverse populations.

Several limitations should be acknowledged. First, chronic pain data were only collected within NHANES from 1999 to 2004, limiting the ability to verify findings across different periods. Second, residual confounding, stemming from unmeasured or undisclosed variables, may persist despite the implementation of regression models, stratified analysis, and sensitivity analysis. In considering the robustness of our findings, we must recognize the potential for unmeasured confounders to influence the relationships we have observed. For instance, genetic predispositions to inflammation and pain variability, psychological stress, and other environmental factors that we were unable to measure may affect both the DII and chronic pain reporting. These unmeasured variables could bias our results in unpredictable ways. For example, if individuals with inherent pro-inflammatory genetics were underrepresented in our study, it might exaggerate the relationship between higher DII scores and chronic pain prevalence. On the other hand, a sample that disproportionately includes individuals with better access to health resources may lead us to understate the dietary impact on chronic pain. It is crucial for future studies to capture these variables more comprehensively to further clarify the nature of these associations and to contribute to a more detailed understanding of the connections between diet, inflammation, and pain. Third, the data utilized in this study originate from a survey conducted among American adults, thereby necessitating further exploration to ascertain its applicability to other demographic groups. Fourth, the DII encompasses 45 food parameters, but only 27 were included in the calculation, potentially introducing bias in estimating dietary inflammation. However, this limitation is expected due to the practical challenges of assessing all food intake comprehensively. Finally, as an inherent limitation of cross-sectional studies, the evidence for causality needs to be improved, necessitating future prospective studies to provide higher-level evidence for the relationship between DII and chronic pain.

The dietary data utilized in our study was derived from the U.S. Department of Agriculture dietary database corresponding to the years 1999–2004. We recognize that during this period, the database did not comprehensively account for a range of food components, including phytochemicals, polyphenols, and other bioactive compounds, which have since been identified as having profound effects on inflammation and oxidative stress. The absence of these data points could potentially affect the estimation of the dietary inflammatory index and, by extension, the associations drawn with chronic pain. This limitation is important to consider when interpreting our findings, as the inclusion of these compounds might refine the DII and provide a more nuanced understanding of the diet-inflammation-pain relationship. Future studies with updated dietary databases that encompass these components may offer more detailed insights.

## Conclusion

In conclusion, our research reveals a U-shaped association between dietary inflammatory index and chronic pain in American adults. These findings may provide a basis for further research into the relationship of diet and chronic pain. Future studies are necessary to explore the potential clinical implications and to determine if modifying dietary inflammation could be beneficial in pain management.

### Supplementary Information

Supplementary Information.

## Data Availability

The datasets analyzed during the current study are available in the National Health and Nutrition Examination Survey repository [http://www.cdc.gov/nchs/nhanes.htm].

## Abbreviations

BMI: Body mass index
CI: Confidence interval
CRP: C-reactive protein
DII: Dietary inflammatory index
IQR: Interquartile range
MPQ: Miscellaneous Pain Questionnaire
NCHS: National Center for Health Statistics
NHANES: National Health and Nutrition Examination Survey
OR: Odds ratio
PIR: Poverty income ratio
RCS: Restricted cubic spline
Ref: Reference
SD: Standard deviation
