# Supplementary material for: Exploring the association between dietary Inflammatory Index and chronic pain in US adults using NHANES 1999–2004
Source: Sci Rep. 2024 Apr 16;14:8726. doi: 10.1038/s41598-024-58030-w (PMC11018766; doi:10.1038/s41598-024-58030-w)
Supplement: Supplementary file 1 — Supplementary Information. [file 41598_2024_58030_MOESM1_ESM.pdf]

Exploring the Association between Dietary inflammatory index and Chronic Pain in US Adults Using NHANES 1999-2004_Lunxue Qing

Table S1. Comparison of basic characteristics between excluded and included populations (age ≥ 20).

Characteristic	Total	Excluded population	Included population	*p*
NO.	15332	12751	2581	
Age(year), Mean (SD)	50.4 (19.5)	50.6 (20.0)	49.6 (16.7)	0.016
Sex, n (%)				0.131
Male	7223 (47.1)	6042 (47.4)	1181 (45.8)	
Female	8109 (52.9)	6709 (52.6)	1400 (54.2)	
Race/ethnicity, n (%)				< 0.001
Non-Hispanic white	7761 (50.6)	6232 (48.9)	1529 (59.2)	
Non-Hispanic black	2916 (19.0)	2474 (19.4)	442 (17.1)	
Mexican American	3380 (22.0)	2961 (23.2)	419 (16.2)	
Others	1275 (8.3)	1084 (8.5)	191 (7.4)	
Marital status, n (%)				0.005
Married or living with a partner	8974 (60.7)	7343 (60.2)	1631 (63.2)	
Living alone	5802 (39.3)	4852 (39.8)	950 (36.8)	
Education level, n (%)				< 0.001
< 9	2456 (16.1)	2160 (17)	296 (11.5)	
9—12	6225 (40.8)	5130 (40.4)	1095 (42.4)	
>12	6592 (43.2)	5402 (42.6)	1190 (46.1)	
PIR, Mean (SD)	2.6 (1.6)	2.6 (1.6)	2.6 (1.6)	0.492
BMI (kg/m^2), Mean (SD)	28.3 (6.2)	28.1 (6.1)	29.2 (6.9)	< 0.001
Physical activity, n (%)				0.031
Sedentary	7190 (47.0)	6039 (47.4)	1151 (44.6)	
Moderate	4121 (26.9)	3395 (26.7)	726 (28.1)	
Vigorous	4000 (26.1)	3296 (25.9)	704 (27.3)	
Smoking status, n (%)				< 0.001
Never	7905 (51.6)	6766 (53.1)	1139 (44.1)	
Former	4089 (26.7)	3366 (26.4)	723 (28)	
Current	3338 (21.8)	2619 (20.5)	719 (27.9)	
Hypertension, n (%)	4041 (26.7)	3215 (25.6)	826 (32)	< 0.001

Diabetes, n (%)	1536 (10.0)	1234 (9.7)	302 (11.7)	0.002
Coronary heart disease, n (%)	707 (4.6)	548 (4.3)	159 (6.2)	< 0.001
Stroke, n (%)	606 (4.0)	503 (4)	103 (4)	0.925
Dietary supplements taken, n (%)	7806 (51.1)	6426 (50.6)	1380 (53.5)	0.007
CRP (mg/dl), Median (IQR)	0.2 (0.1, 0.5)	0.2 (0.1, 0.5)	0.2 (0.1, 0.6)	< 0.001
DII, Mean (SD)	0.9 (1.9)	0.9 (1.9)	1.0 (1.9)	0.006
Chronic pain, n (%)	2173 (60.7)	633(63.5)	1540 (59.7)	0.036

BMI: body mass index; CRP: C-reactive protein; DII: dietary inflammatory index. PIR: poverty income ratio

Table S2. Nutrient intake according to the quartiles of the DII

Variables	Total	Dietary inflammatory index					
		Q1 (-4.98–-0.2)	Q2 (-0.21 – -1.3)	Q3 (1.31 – -2.49)	Q4 (2.5 – 4.69)	anti-inflammatory (<0)	pro-inflammatory (>0)
n	2671	635	642	641	663	719	1862
Dietary inflammatory index, Mean (SD)	1.03 (1.88)	-1.61 (1.00)	0.57 (0.45)	1.88 (0.35)	3.19 (0.47)	-1.44 (1.05)	1.99 (1.10)
Energy, kcal, Mean (SD)	2144.75 (1079.60)	3001.13 (1333.14)	2282.98 (792.93)	1950.76 (710.18)	1378.24 (630.13)	2942.37 (1290.73)	1836.75 (795.22)
Protein, g, Mean (SD)	79.07 (43.94)	113.00 (53.89)	86.04 (33.81)	70.48 (28.82)	48.12 (25.57)	110.51 (52.27)	66.93 (33.07)
Carbohydrate (g), Mean (SD)	264.80 (140.15)	367.80 (167.06)	274.33 (109.34)	241.46 (103.72)	179.51 (98.82)	359.73 (163.28)	228.15 (110.08)
Fiber (g), Mean (SD)	30.67 (37.66)	50.09 (51.00)	33.21 (34.68)	23.10 (27.45)	16.94 (23.08)	49.33 (50.26)	23.47 (28.38)
Total fat (g), Mean (SD)	67.68 (48.58)	91.44 (63.41)	71.95 (44.71)	63.81 (38.17)	44.55 (29.86)	89.92 (61.95)	59.10 (39.07)
Saturated fatty acid (g), Mean (SD)	27.30 (17.83)	36.83 (23.19)	29.19 (15.21)	24.82 (13.81)	18.74 (12.07)	36.47 (22.54)	23.76 (14.13)
Mono unsaturated fatty acid (g), Mean (SD)	26.94 (18.13)	37.33 (22.71)	28.86 (16.56)	24.70 (13.64)	17.27 (11.65)	36.77 (22.14)	23.14 (14.65)
Polyunsaturated fatty acid (g), Mean (SD)	84.29 (181.05)	123.59 (247.16)	90.73 (158.30)	67.35 (145.72)	56.77 (147.99)	124.45 (244.92)	68.78 (146.42)
Cholesterol (mg), Mean (SD)	224.65 (244.93)	275.40 (291.08)	246.23 (271.61)	221.96 (209.26)	157.75 (177.44)	273.45 (290.48)	205.81 (222.13)
Vitamin E (mg), Mean (SD)	244.94 (662.78)	472.83 (973.26)	254.11 (617.58)	172.26 (527.81)	88.07 (294.13)	448.78 (931.21)	166.23 (502.17)
Vitamin A (RE), Mean (SD)	573.58 (750.24)	993.75 (1040.97)	607.57 (673.91)	477.81 (628.97)	230.82 (191.38)	969.00 (1052.08)	420.89 (519.17)
Beta carotene(μg), Mean (SD)	1336.62 (2920.41)	2881.92 (4780.85)	1357.70 (2460.19)	782.22 (1248.81)	372.17 (678.92)	2716.31 (4563.50)	803.86 (1664.23)
Thiamine (mg), Mean (SD)	1.64 (1.01)	2.49 (1.27)	1.78 (0.78)	1.38 (0.56)	0.94 (0.50)	2.43 (1.25)	1.33 (0.69)
Riboflavin (mg), Mean (SD)	7.14 (10.80)	11.67 (15.65)	8.07 (10.55)	5.11 (6.93)	3.88 (5.57)	11.44 (15.22)	5.48 (7.90)

Niacin (mg), Mean (SD)	17.38 (14.84)	25.09 (19.98)	19.30 (14.52)	15.80 (10.14)	9.65 (7.08)	24.38 (19.52)	14.68 (11.50)
Vitamin B6 (mg), Mean (SD)	92.15 (191.36)	172.00 (294.27)	100.08 (177.25)	57.12 (113.67)	41.87 (83.51)	167.67 (286.11)	62.99 (127.01)
Folate (µg), Mean (SD)	317.11 (266.47)	512.09 (360.20)	345.24 (237.10)	259.37 (150.67)	158.94 (102.90)	495.41 (349.77)	248.26 (184.98)
Vitamin C (mg), Mean (SD)	68.93 (104.21)	125.53 (151.56)	69.99 (87.85)	53.58 (80.00)	28.53 (41.55)	119.98 (146.41)	49.22 (73.41)
Magnesium (mg), Mean (SD)	271.81 (146.35)	439.79 (157.24)	290.61 (79.66)	220.39 (63.89)	142.42 (54.48)	426.94 (154.45)	211.90 (87.19)
Iron (mg), Mean (SD)	15.01 (9.63)	23.63 (12.22)	16.30 (7.44)	12.26 (4.79)	8.18 (3.97)	22.92 (11.83)	11.96 (6.41)
Zinc (mg), Mean (SD)	11.48 (9.49)	17.26 (9.92)	12.26 (6.44)	10.06 (12.09)	6.56 (4.08)	16.78 (9.62)	9.43 (8.61)
Selenium (mg), Mean (SD)	105.18 (65.83)	152.39 (88.03)	112.97 (51.88)	93.72 (40.86)	63.51 (34.45)	149.50 (85.82)	88.07 (46.00)
Caffeine (mg), Mean (SD)	195.96 (265.96)	225.34 (295.25)	188.63 (290.65)	204.42 (265.43)	166.74 (201.19)	218.47 (290.43)	187.26 (255.44)
Alcohol (g), Mean (SD)	10.93 (35.45)	17.21 (52.29)	13.82 (36.32)	10.19 (27.08)	2.85 (13.44)	16.67 (50.85)	8.72 (26.96)
n-6 Fatty acids (g), Mean (SD)	9.94 (10.59)	14.81 (14.63)	11.16 (10.00)	8.80 (8.12)	5.19 (4.89)	14.60 (14.30)	8.14 (8.07)
n-3 Fatty acids (g), Mean (SD)	6.53 (8.92)	10.24 (11.97)	6.94 (9.28)	5.75 (7.00)	3.33 (4.24)	9.95 (11.73)	5.21 (7.14)
Vitamin B12 (µg), Mean (SD)	5.40 (8.87)	8.34 (14.65)	5.82 (6.23)	4.57 (5.39)	2.98 (4.54)	8.23 (14.39)	4.31 (4.99)

Table S3. The relationship between DII and chronic pain in individuals who do not have excessively energy intake[a]

Outcome	Total	Chronic pain (n, %)	Crude Model		Model 1		Model 2		Model 3	
			OR (95% CI)	*p*-Value	OR (95% CI)	*p*-Value	OR (95% CI)	*p*-Value	OR (95% CI)	*p*-Value
DII	2500	1493(59.7)	1.09 (1.03–1.05)	0.006	1.1 (1.04–1.17)	0.001	1.08 (1.02–1.14)	0.034	1.05 (0.99–1.11)	0.109
DII (quartile)										
Q1 (≤ -0.14)	591	332 (56.2)	1(Ref)		1(Ref)		1(Ref)		1(Ref)	
Q2 (-0.15–1.31)	639	359 (56.2)	1.02 (0.72–1.44)	0.903	1.05 (0.77–1.45)	0.738	1.01 (0.73–1.4)	0.939	0.93 (0.67–1.28)	0.636
Q3 (1.32–2.48)	638	390 (61.1)	1.4 (1.02–1.92)	0.04	1.5 (1.09–2.06)	0.014	1.39 (1–1.95)	0.051	1.3 (0.92–1.82)	0.131
Q4 (≥ 2.49)	632	412 (65.2)	1.62 (1.19–2.2)	0.003	1.81 (1.35–2.44)	<0.001	1.64 (1.22–2.2)	0.002	1.41 (1.05–1.9)	0.023
Trend test				<0.001		<0.001		<0.001		0.005

CI, confidence interval; DII: dietary inflammatory index; OR, odd ratio; Ref: reference.

[a] Less than 500 kcal or more than 5000 kcal is considered as excessively energy intake.

All analyses have been weighted to account for the survey's complex sampling design.

Model I: Adjusted for age and sex.

Model II: Adjusted for age, sex, race, marital status, poverty income ratio, and education level.

Model III: Adjusted for all these variables, including age, sex race, marital status, poverty income ratio, education level, body mass index, physical activity, smoking status, coronary heart disease, stroke, dietary supplements taken, hypertension, diabetes mellitus and C-reactive protein.

Subgroup	No of participants	OR (95%CI)	P for interaction
**Sex**			**0.65**
Male	1181	1.03(0.97,1.1)	
Female	1400	1.05(0.99,1.12)	
**BMI**			**0.486**
< 25	728	1.00(0.93,1.08)	
≥ 25	1853	1.06(1.01,1.12)	
**Hypertension**			**0.823**
Yes	826	1.08(1.00,1.17)	
No	1755	1.03(0.97,1.08)	
**Diabetes**			**0.142**
Yes	302	0.95(0.83,1.09)	
No	2279	1.05(1.01,1.11)	

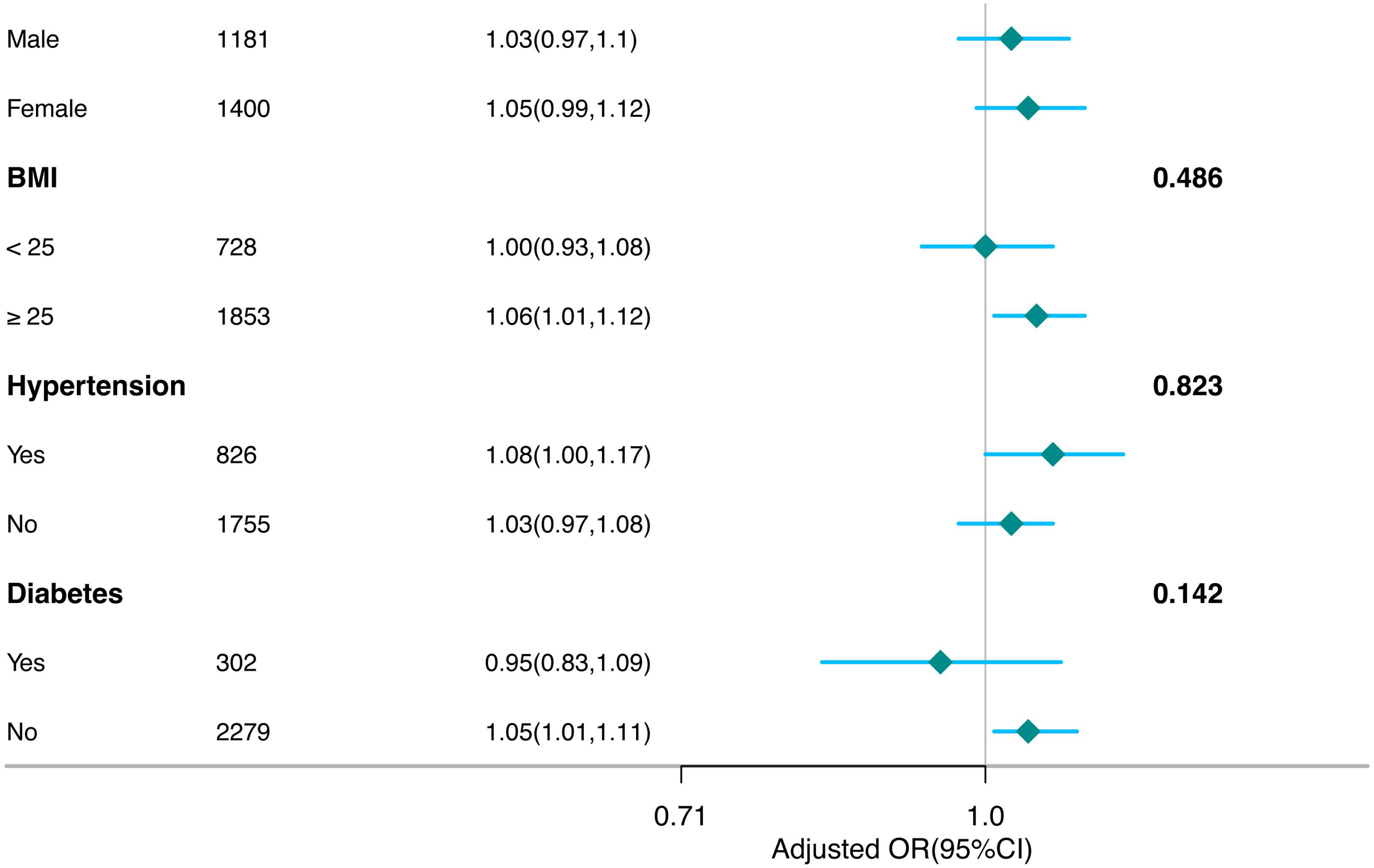

Figure S1. Weighted analysis of the interaction between dietary inflammatory index and chronic pain.

Abbreviations: BMI: body mass index; CI: confidence interval; OR: odd ratio;

Note: P for interaction represents the likelihood of interaction between the variable and dietary inflammatory index.

Each stratified factor was analyzed for its interaction with the dietary inflammatory index on chronic pain, adjusting for all other variables, including age, sex, marital status, race/ethnicity, education level, household income, smoking status, physical activity, hypertension, diabetes, stroke, coronary heart disease, body mass index, energy expenditure, protein consumption, carbohydrate consumption, fat consumption, dietary supplements taken, and C-reactive protein, with the exception of the stratification variable itself.

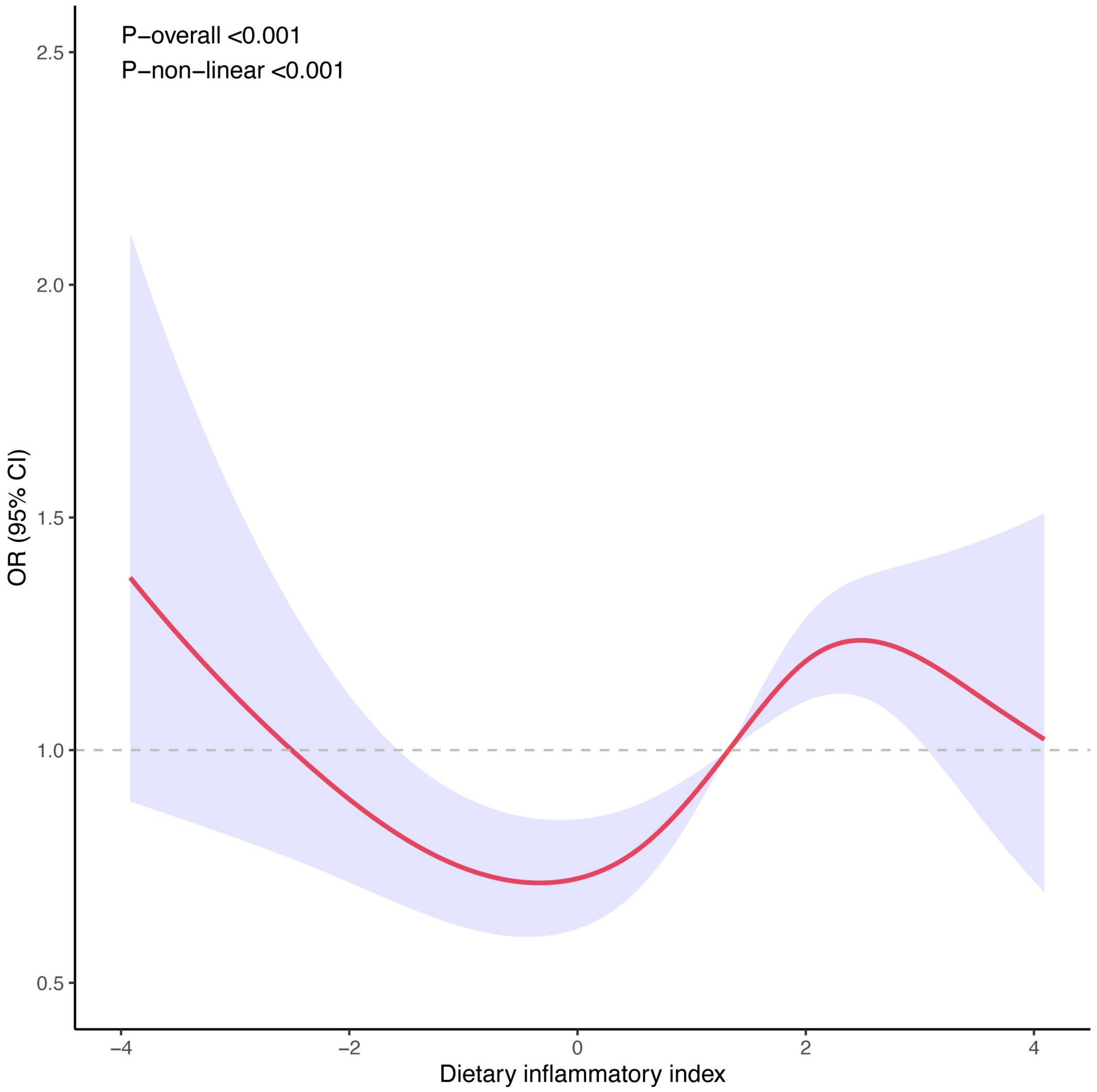

Figure S2. Non-linear association between the dietary inflammatory index and chronic pain in individuals who do not have excessively energy intake.

Note: The curve presented in the figure is based on a weighted restricted cubic spline model to illustrate the non-linear association between the dietary inflammatory index and chronic pain in individuals who do not have excessively energy intake. Adjusted for age, sex, race, marital, poverty income ratio, education level, body mass index, physical activity, smoking status, coronary heart disease, stroke, hypertension, diabetes mellitus, dietary supplements, and C-reactive protein levels.

Reporting checklist for cross sectional study.

Based on the STROBE cross sectional guidelines.

Instructions to authors

Complete this checklist by entering the page numbers from your manuscript where readers will find each of the items listed below.

Your article may not currently address all the items on the checklist. Please modify your text to include the missing information. If you are certain that an item does not apply, please write "n/a" and provide a short explanation.

Upload your completed checklist as an extra file when you submit to a journal.

In your methods section, say that you used the STROBE cross sectionalreporting guidelines, and cite them as:

von Elm E, Altman DG, Egger M, Pocock SJ, Gotzsche PC, Vandenbroucke JP. The Strengthening the Reporting of Observational Studies in Epidemiology (STROBE) Statement: guidelines for reporting observational studies.

		Reporting Item	Page Number
**Title and abstract**			
Title	#1a	Indicate the study's design with a commonly used term in the title or the abstract	1
Abstract	#1b	Provide in the abstract an informative and balanced summary of what was done and what was found	1–2
**Introduction**			

Background / rationale	#2	Explain the scientific background and rationale for the investigation being reported	3–5
Objectives	#3	State specific objectives, including any prespecified hypotheses	5
**Methods**			
Study design	#4	Present key elements of study design early in the paper	5–6
Setting	#5	Describe the setting, locations, and relevant dates, including periods of recruitment, exposure, follow-up, and data collection	5–6
Eligibility criteria	#6a	Give the eligibility criteria, and the sources and methods of selection of participants.	6
	#7	Clearly define all outcomes, exposures, predictors, potential confounders, and effect modifiers. Give diagnostic criteria, if applicable	6 - 9
Data sources / measurement	#8	For each variable of interest give sources of data and details of methods of assessment (measurement). Describe comparability of assessment methods if there is more than one group. Give information separately for for exposed and unexposed groups if applicable.	6 - 9
Bias	#9	Describe any efforts to address potential sources of bias	11
Study size	#10	Explain how the study size was arrived at	11
Quantitative variables	#11	Explain how quantitative variables were handled in the analyses. If applicable, describe which groupings were chosen, and why	9–10
Statistical methods	#12a	Describe all statistical methods, including those used to control for confounding	9–10

Statistical methods	#12b	Describe any methods used to examine subgroups and interactions	10
Statistical methods	#12c	Explain how missing data were addressed	11
Statistical methods	#12d	If applicable, describe analytical methods taking account of sampling strategy	n/a, not relevant.
Statistical methods	#12e	Describe any sensitivity analyses	11
**Results**			
Participants	#13a	Report numbers of individuals at each stage of study—eg numbers potentially eligible, examined for eligibility, confirmed eligible, included in the study, completing follow-up, and analysed. Give information separately for for exposed and unexposed groups if applicable.	11
Participants	#13b	Give reasons for non-participation at each stage	11
Participants	#13c	Consider use of a flow diagram	Figure 1
Descriptive data	#14a	Give characteristics of study participants (eg demographic, clinical, social) and information on exposures and potential confounders. Give information separately for exposed and unexposed groups if applicable.	Table 1
Descriptive data	#14b	Indicate number of participants with missing data for each variable of interest	11
Outcome data	#15	Report numbers of outcome events or summary measures. Give information separately for exposed and unexposed groups if applicable.	11–12
Main results	#16a	Give unadjusted estimates and, if applicable, confounder-adjusted estimates and their precision (eg, 95% confidence interval). Make clear which confounders were adjusted for and why they were included	12–13

Main results	#16b	Report category boundaries when continuous variables were categorized	910
Main results	#16c	If relevant, consider translating estimates of relative risk into absolute risk for a meaningful time period	n/a, not relevant.
Other analyses	#17	Report other analyses done—e.g., analyses of subgroups and interactions, and sensitivity analyses	13–14
**Discussion**			
Key results	#18	Summarise key results with reference to study objectives	14
Limitations	#19	Discuss limitations of the study, taking into account sources of potential bias or imprecision. Discuss both direction and magnitude of any potential bias.	18–20
Interpretation	#20	Give a cautious overall interpretation considering objectives, limitations, multiplicity of analyses, results from similar studies, and other relevant evidence.	18-20
Generalisability	#21	Discuss the generalisability (external validity) of the study results	20
**Other Information**			
Funding	#22	Give the source of funding and the role of the funders for the present study and, if applicable, for the original study on which the present article is based	22

None The STROBE checklist is distributed under the terms of the Creative Commons Attribution License CC-BY. This checklist can be completed online using https://www.goodreports.org/, a tool made by the EQUATOR Network in collaboration with Penelope.ai
